# μ-1,4-Bis(pyridin-4-ylmeth­yl)piperazine-κ^2^ *N*:*N*′-bis­[aqua­bis­(3-bromo-5-carb­oxy­benzoato-κ*O* ^1^)copper(II)]

**DOI:** 10.1107/S1600536812000979

**Published:** 2012-01-14

**Authors:** Jodi L. Meyer, Robert L. LaDuca

**Affiliations:** a Lyman Briggs College, Department of Chemistry, Michigan State University, East Lansing, MI 48825, USA

## Abstract

In the title compound, [Cu_2_(C_8_H_4_BrO_4_)_4_(C_16_H_20_N_4_)(H_2_O)_2_], slightly distorted square-planar-coordinated Cu^II^ ions are bound by one aqua ligand and two monodentate 3-bromo-5-carb­oxy­benzoate anions, and linked into a centrosymmetric dinuclear mol­ecule by a bridging 1,4-bis­(pyridin-4-ylmeth­yl)piperazine (4-bpmp) ligand. In the crystal, mol­ecules are connected into a supra­molecular two-dimensional network parallel to (131) *via* O—H⋯O hydrogen bonds involving the aqua ligands and 3-bromo-5-carb­oxy­benzoate carboxyl­ate groups.

## Related literature

For other copper coordination polymers containing 4-bpmp ligands, see: Sposato *et al.* (2010 ▶); Gandolfo & LaDuca (2011 ▶).
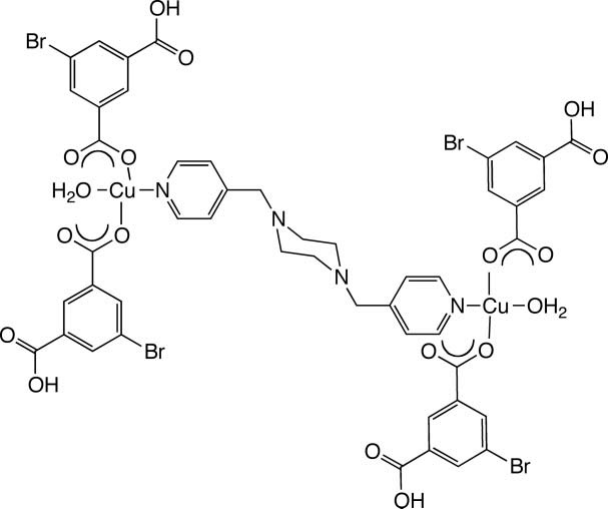


## Experimental

### 

#### Crystal data

[Cu_2_(C_8_H_4_BrO_4_)_4_(C_16_H_20_N_4_)(H_2_O)_2_]
*M* *_r_* = 1407.56
Triclinic, 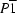

*a* = 7.136 (2) Å
*b* = 11.925 (4) Å
*c* = 16.577 (5) Å
α = 74.458 (3)°
β = 86.358 (4)°
γ = 72.908 (3)°
*V* = 1299.0 (7) Å^3^
*Z* = 1
Mo *K*α radiation
μ = 3.97 mm^−1^
*T* = 173 K
0.37 × 0.26 × 0.11 mm

#### Data collection

Bruker APEXII CCD diffractometer
Absorption correction: multi-scan (*SADABS*; Sheldrick, 1996 ▶) *T*_min_ = 0.320, *T*_max_ = 0.674
20701 measured reflections
4723 independent reflections
4142 reflections with *I* > 2σ(*I*)
*R*_int_ = 0.025

#### Refinement

*R*[*F* ^2^ > 2σ(*F* ^2^)] = 0.034
*wR*(*F* ^2^) = 0.092
*S* = 1.05
4723 reflections
355 parameters
5 restraints
H atoms treated by a mixture of independent and constrained refinement
Δρ_max_ = 0.73 e Å^−3^
Δρ_min_ = −0.60 e Å^−3^

### 

Data collection: *APEX2* (Bruker, 2006 ▶); cell refinement: *SAINT* (Bruker, 2006 ▶); data reduction: *SAINT*; program(s) used to solve structure: *SHELXS97* (Sheldrick, 2008 ▶); program(s) used to refine structure: *SHELXL97* (Sheldrick, 2008 ▶); molecular graphics: *CrystalMaker* (Palmer, 2007 ▶); software used to prepare material for publication: *SHELXL97*.

## Supplementary Material

Crystal structure: contains datablock(s) I, global. DOI: 10.1107/S1600536812000979/lh5391sup1.cif

Structure factors: contains datablock(s) I. DOI: 10.1107/S1600536812000979/lh5391Isup2.hkl

Additional supplementary materials:  crystallographic information; 3D view; checkCIF report

## Figures and Tables

**Table 1:** Hydrogen-bond geometry (Å, °)

*D*—H⋯*A*	*D*—H	H⋯*A*	*D*⋯*A*	*D*—H⋯*A*
O3—H3*A*⋯O7^i^	0.84 (2)	1.98 (3)	2.763 (4)	155 (4)
O8—H8*C*⋯O4^ii^	0.84 (2)	1.81 (2)	2.629 (4)	166 (4)
O9—H9*A*⋯O2^iii^	0.83 (2)	1.82 (2)	2.635 (3)	169 (4)
O9—H9*B*⋯O6^iii^	0.82 (2)	1.83 (2)	2.647 (3)	171 (4)

Symmetry codes: (i) 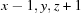; (ii) 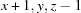; (iii) 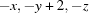.

**Fig. 1.**
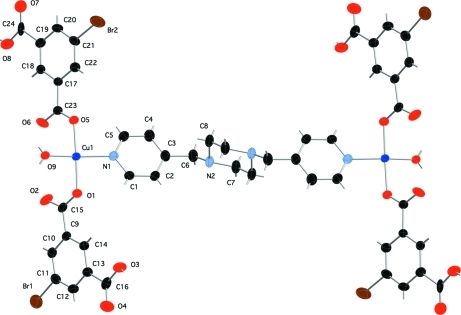
A single {[Cu(H2O)(HBrip)2]2(4-bpmp)} molecule, showing 50% probability ellipsoids and partial atom numbering scheme. Hydrogen atom positions are shown as grey sticks. Color codes: dark blue Cu, red O, light blue N, black C, brown Br. The unlabeled atoms are related by the symmetry operator (-x + 2, -y + 1, -z + 1).

**Fig. 2.**
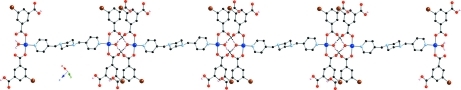
A supramolecular chain of {[Cu(H2O)(HBrip)2]2(4-bpmp)} molecules, with O—H···O hydrogen bonding shown as dashed lines.

**Fig. 3.**
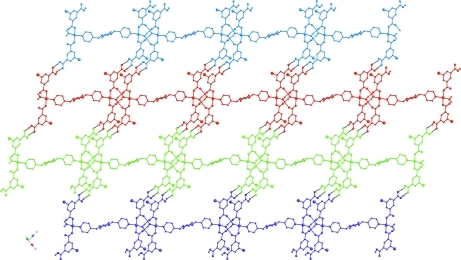
A supramolecular layer formed from {[Cu(H2O)(HBrip)2]2(4-bpmp)} supramolecular chains, with interlayer O—H···O hydrogen bonding shown as dashed lines.

**Fig. 4.**
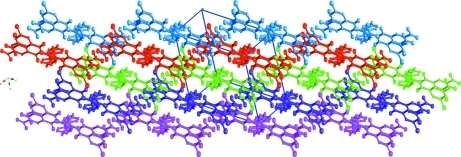
Packing diagram for the title compound.

## supplementary crystallographic information

### Comment

Recently we have been investigating divalent copper coordination polymers containing tethering bis(pyridin-4-ylmethyl)piperazine (4-bpmp) ligands. (Sposato, *et al.*, 2010; Gandolfo & LaDuca, 2011). The title compound was obtained upon an attempt to prepare a copper 4-bpmp coordination polymer incorporating 5-bromoisophthalate (Brip).

The asymmetric unit of the title compound contains a Cu^II^ ion, an aqua ligand, two HBrip ligands, and half of a 4-bpmp ligand whose chair conformation piperazinyl ring centroid lies on a crystallographic inversion center. The Cu^II^ ion is coordinated in a square planar manner, with carboxylate O atom donors from two monodentate HBrip ligands in *trans* positions. The aqua ligand and a pyridyl N atom donor from a 4-bpmp ligand occupy the other two *trans* positions. Two [Cu(H_2_O)(HBrip)_2_] fragments are connected into a {[Cu(H_2_O)(HBrip)_2_]_2_(4-bpmp)} dinuclear molecular species (Fig. 1) by a tethering 4-bpmp ligand.

Individual {[Cu(H_2_O)(HBrip)_2_]_2_(4-bpmp)} molecules aggregate into supramolecular chains by means of O—H···O hydrogen bonding between aqua ligands and unligated O atoms belonging to the ligating HBrip monodentate carboxylate groups (Fig. 2). The chains are arranged parallel to the [1 0 1] crystal direction. In turn these supramolecular chains are connected into supramolecular layers aligned parallel to the (1 3 1) crystal planes *via* O—H···O hydrogen bonding between protonated HBrip carboxylate groups (Fig. 3). Crystal packing forces are responsible for the aggregation of the supramolecular layers into the full crystal structure of the title compound (Fig. 4).

### Experimental

All starting materials were obtained commercially. A mixture of Cu(NO_3_)_2_.2.5H_2_O (51 mg, 0.22 mmol), 4-bpmp (74 mg, 0.28 mmol), H_2_Brip (68 mg, 0.28 mmol), 0.5 ml concentrated nitric acid and 10.0 g water (550 mmol) was placed into a 23 ml Teflon-lined Parr acid digestion bomb, which was then heated under autogenous pressure at 393 K for 24 h. Blue blocks of the title compound were isolated.

### Refinement

All H atoms bound to C atoms were placed in calculated positions, with C—H = 0.95 Å, and refined in riding mode with *U*_iso_ = 1.2*U*_eq_(C). The H atoms bound to the aqua ligand O atom and carboxylate O atoms were found in a difference Fourier map, restrained with O—H = 0.85 Å and refined with *U*_iso_ =1.2*U*_eq_(O).

### Crystal data

**Table:**

[Cu_2_(C_8_H_4_BrO_4_)_4_(C_16_H_20_N_4_)(H_2_O)_2_]	*Z* = 1
*M**_r_* = 1407.56	*F*(000) = 698
Triclinic, *P*1	*D*_x_ = 1.799 Mg m^−^^3^
Hall symbol: -P 1	Mo *K*α radiation, λ = 0.71073 Å
*a* = 7.136 (2) Å	Cell parameters from 9934 reflections
*b* = 11.925 (4) Å	θ = 2.5–25.3°
*c* = 16.577 (5) Å	µ = 3.97 mm^−^^1^
α = 74.458 (3)°	*T* = 173 K
β = 86.358 (4)°	Chunk, blue
γ = 72.908 (3)°	0.37 × 0.26 × 0.11 mm
*V* = 1299.0 (7) Å^3^	

### Data collection

**Table:**

Bruker APEXII CCD diffractometer	4723 independent reflections
Radiation source: fine-focus sealed tube	4142 reflections with *I* > 2σ(*I*)
graphite	*R*_int_ = 0.025
φ and ω scans	θ_max_ = 25.3°, θ_min_ = 2.0°
Absorption correction: multi-scan (*SADABS*; Sheldrick, 1996)	*h* = −8→8
*T*_min_ = 0.320, *T*_max_ = 0.674	*k* = −14→14
20701 measured reflections	*l* = −19→19

### Refinement

**Table:**

Refinement on *F*^2^	Primary atom site location: structure-invariant direct methods
Least-squares matrix: full	Secondary atom site location: difference Fourier map
*R*[*F*^2^ > 2σ(*F*^2^)] = 0.034	Hydrogen site location: inferred from neighbouring sites
*wR*(*F*^2^) = 0.092	H atoms treated by a mixture of independent and constrained refinement
*S* = 1.05	*w* = 1/[σ^2^(*F*_o_^2^) + (0.0434*P*)^2^ + 2.2722*P*] where *P* = (*F*_o_^2^ + 2*F*_c_^2^)/3
4723 reflections	(Δ/σ)_max_ < 0.001
355 parameters	Δρ_max_ = 0.73 e Å^−^^3^
5 restraints	Δρ_min_ = −0.59 e Å^−^^3^

### Special details

**Table:**

Geometry. All e.s.d.'s (except the e.s.d. in the dihedral angle between two l.s. planes) are estimated using the full covariance matrix. The cell e.s.d.'s are taken into account individually in the estimation of e.s.d.'s in distances, angles and torsion angles; correlations between e.s.d.'s in cell parameters are only used when they are defined by crystal symmetry. An approximate (isotropic) treatment of cell e.s.d.'s is used for estimating e.s.d.'s involving l.s. planes.
Refinement. Refinement of *F*^2^ against ALL reflections. The weighted *R*-factor *wR* and goodness of fit *S* are based on *F*^2^, conventional *R*-factors *R* are based on *F*, with *F* set to zero for negative *F*^2^. The threshold expression of *F*^2^ > σ(*F*^2^) is used only for calculating *R*-factors(gt) *etc*. and is not relevant to the choice of reflections for refinement. *R*-factors based on *F*^2^ are statistically about twice as large as those based on *F*, and *R*- factors based on ALL data will be even larger.

### Fractional atomic coordinates and isotropic or equivalent isotropic displacement parameters (Å^2^)

**Table:**

	*x*	*y*	*z*	*U*_iso_*/*U*_eq_	
Cu1	0.24171 (6)	0.87361 (3)	0.11476 (2)	0.02507 (11)	
Br1	−0.40313 (7)	1.48207 (3)	0.24403 (3)	0.05057 (14)	
Br2	0.86689 (8)	0.43000 (4)	−0.07443 (3)	0.05927 (15)	
O1	0.0823 (3)	0.9602 (2)	0.18966 (14)	0.0362 (6)	
O2	0.0127 (4)	1.1404 (2)	0.09417 (14)	0.0400 (6)	
O3	−0.1644 (4)	0.9305 (2)	0.47763 (16)	0.0431 (6)	
H3A	−0.210 (6)	0.899 (4)	0.5231 (17)	0.052*	
O4	−0.3480 (4)	1.0979 (2)	0.50793 (17)	0.0510 (7)	
O5	0.3990 (3)	0.7968 (2)	0.03553 (13)	0.0317 (5)	
O6	0.3406 (4)	0.9682 (2)	−0.06839 (16)	0.0389 (6)	
O7	0.8012 (4)	0.7817 (2)	−0.36611 (16)	0.0408 (6)	
O8	0.6280 (4)	0.9548 (2)	−0.34293 (16)	0.0432 (6)	
H8C	0.636 (6)	0.990 (4)	−0.3933 (14)	0.052*	
O9	0.0062 (3)	0.8750 (2)	0.06054 (13)	0.0280 (5)	
H9A	0.009 (5)	0.861 (3)	0.0143 (15)	0.034*	
H9B	−0.097 (4)	0.925 (3)	0.067 (2)	0.034*	
N1	0.4710 (4)	0.8158 (2)	0.19417 (16)	0.0268 (6)	
N2	0.9406 (5)	0.6125 (2)	0.43776 (17)	0.0346 (7)	
C1	0.4470 (5)	0.8125 (3)	0.2762 (2)	0.0380 (8)	
H1	0.3177	0.8370	0.2963	0.046*	
C2	0.6023 (6)	0.7753 (4)	0.3317 (2)	0.0448 (10)	
H2	0.5788	0.7726	0.3891	0.054*	
C3	0.7919 (5)	0.7418 (3)	0.3041 (2)	0.0331 (8)	
C4	0.8168 (5)	0.7434 (3)	0.2208 (2)	0.0353 (8)	
H4	0.9449	0.7192	0.1995	0.042*	
C5	0.6543 (5)	0.7804 (3)	0.1680 (2)	0.0315 (7)	
H5	0.6746	0.7807	0.1108	0.038*	
C6	0.9650 (6)	0.7027 (3)	0.3631 (2)	0.0399 (9)	
H6A	1.0860	0.6686	0.3345	0.048*	
H6B	0.9793	0.7743	0.3788	0.048*	
C7	1.0859 (6)	0.5919 (3)	0.5029 (2)	0.0418 (9)	
H7A	1.0776	0.6696	0.5158	0.050*	
H7B	1.2195	0.5599	0.4827	0.050*	
C8	0.9526 (6)	0.4977 (3)	0.4194 (2)	0.0400 (9)	
H8B	1.0845	0.4643	0.3984	0.048*	
H8A	0.8549	0.5117	0.3753	0.048*	
C9	−0.1060 (4)	1.1335 (3)	0.23134 (19)	0.0254 (7)	
C10	−0.1898 (5)	1.2583 (3)	0.2111 (2)	0.0284 (7)	
H10	−0.1788	1.3066	0.1562	0.034*	
C11	−0.2900 (5)	1.3120 (3)	0.2721 (2)	0.0317 (7)	
C12	−0.3085 (5)	1.2433 (3)	0.3524 (2)	0.0319 (7)	
H12	−0.3785	1.2812	0.3934	0.038*	
C13	−0.2238 (5)	1.1186 (3)	0.3724 (2)	0.0288 (7)	
C14	−0.1224 (5)	1.0639 (3)	0.3121 (2)	0.0277 (7)	
H14	−0.0639	0.9784	0.3260	0.033*	
C15	0.0040 (5)	1.0757 (3)	0.1651 (2)	0.0279 (7)	
C16	−0.2487 (5)	1.0454 (3)	0.46047 (19)	0.0294 (7)	
C17	0.5456 (4)	0.7858 (3)	−0.09479 (19)	0.0239 (6)	
C18	0.5706 (4)	0.8441 (3)	−0.17780 (19)	0.0240 (6)	
H18	0.5100	0.9288	−0.1986	0.029*	
C19	0.6838 (5)	0.7784 (3)	−0.22993 (19)	0.0268 (7)	
C20	0.7747 (5)	0.6545 (3)	−0.1999 (2)	0.0314 (7)	
H20	0.8527	0.6092	−0.2354	0.038*	
C21	0.7489 (5)	0.5990 (3)	−0.1172 (2)	0.0316 (7)	
C22	0.6352 (5)	0.6626 (3)	−0.0643 (2)	0.0292 (7)	
H22	0.6187	0.6223	−0.0077	0.035*	
C23	0.4175 (4)	0.8580 (3)	−0.03952 (19)	0.0260 (7)	
C24	0.7112 (5)	0.8415 (3)	−0.32309 (19)	0.0252 (7)	

### Atomic displacement parameters (Å^2^)

**Table:**

	*U*^11^	*U*^22^	*U*^33^	*U*^12^	*U*^13^	*U*^23^
Cu1	0.0300 (2)	0.0234 (2)	0.01558 (19)	−0.00011 (16)	0.00140 (15)	−0.00329 (15)
Br1	0.0652 (3)	0.0247 (2)	0.0584 (3)	−0.00651 (18)	−0.0128 (2)	−0.00904 (17)
Br2	0.0773 (3)	0.0264 (2)	0.0558 (3)	0.0053 (2)	0.0118 (2)	−0.00513 (18)
O1	0.0357 (13)	0.0405 (15)	0.0253 (12)	0.0039 (11)	−0.0002 (10)	−0.0128 (11)
O2	0.0579 (17)	0.0459 (15)	0.0229 (13)	−0.0234 (13)	0.0081 (11)	−0.0123 (11)
O3	0.0610 (17)	0.0323 (14)	0.0280 (13)	−0.0070 (12)	0.0144 (12)	−0.0047 (11)
O4	0.0648 (19)	0.0323 (14)	0.0424 (16)	−0.0003 (13)	0.0116 (14)	−0.0051 (12)
O5	0.0351 (13)	0.0334 (13)	0.0207 (11)	0.0001 (10)	0.0014 (9)	−0.0084 (10)
O6	0.0384 (14)	0.0260 (13)	0.0429 (15)	0.0009 (11)	0.0127 (11)	−0.0074 (11)
O7	0.0484 (16)	0.0356 (14)	0.0350 (14)	−0.0123 (12)	0.0024 (12)	−0.0037 (12)
O8	0.0585 (17)	0.0375 (15)	0.0266 (13)	−0.0093 (13)	0.0128 (12)	−0.0046 (11)
O9	0.0304 (12)	0.0299 (12)	0.0183 (11)	−0.0006 (10)	0.0018 (9)	−0.0065 (9)
N1	0.0349 (15)	0.0215 (13)	0.0198 (13)	−0.0043 (11)	0.0017 (11)	−0.0030 (10)
N2	0.0468 (18)	0.0238 (14)	0.0284 (15)	−0.0024 (13)	−0.0162 (13)	−0.0037 (11)
C1	0.041 (2)	0.040 (2)	0.0226 (17)	0.0046 (16)	0.0018 (15)	−0.0093 (15)
C2	0.054 (2)	0.044 (2)	0.0227 (17)	0.0095 (18)	−0.0071 (16)	−0.0104 (16)
C3	0.044 (2)	0.0198 (16)	0.0297 (18)	−0.0044 (15)	−0.0068 (15)	0.0001 (13)
C4	0.0334 (19)	0.038 (2)	0.0301 (18)	−0.0098 (16)	0.0004 (14)	−0.0019 (15)
C5	0.0361 (19)	0.0344 (18)	0.0214 (16)	−0.0102 (15)	0.0034 (14)	−0.0038 (14)
C6	0.047 (2)	0.0309 (19)	0.037 (2)	−0.0070 (17)	−0.0125 (17)	−0.0034 (15)
C7	0.053 (2)	0.0287 (18)	0.041 (2)	−0.0057 (17)	−0.0224 (18)	−0.0069 (16)
C8	0.054 (2)	0.0286 (18)	0.0341 (19)	−0.0036 (17)	−0.0204 (17)	−0.0069 (15)
C9	0.0234 (15)	0.0309 (17)	0.0232 (15)	−0.0074 (13)	0.0001 (12)	−0.0096 (13)
C10	0.0292 (17)	0.0306 (17)	0.0266 (16)	−0.0105 (14)	−0.0031 (13)	−0.0064 (13)
C11	0.0321 (18)	0.0251 (17)	0.0393 (19)	−0.0073 (14)	−0.0041 (15)	−0.0111 (14)
C12	0.0318 (18)	0.0355 (19)	0.0315 (18)	−0.0092 (15)	0.0047 (14)	−0.0154 (15)
C13	0.0295 (17)	0.0301 (17)	0.0275 (17)	−0.0085 (14)	0.0030 (13)	−0.0096 (14)
C14	0.0279 (16)	0.0289 (17)	0.0250 (16)	−0.0056 (14)	0.0019 (13)	−0.0080 (13)
C15	0.0260 (16)	0.0378 (19)	0.0232 (16)	−0.0100 (14)	−0.0013 (13)	−0.0124 (14)
C16	0.0289 (17)	0.0364 (19)	0.0164 (15)	−0.0131 (15)	−0.0018 (13)	0.0083 (14)
C17	0.0228 (15)	0.0260 (16)	0.0231 (15)	−0.0058 (13)	0.0016 (12)	−0.0087 (13)
C18	0.0227 (15)	0.0258 (16)	0.0236 (15)	−0.0071 (13)	−0.0002 (12)	−0.0064 (13)
C19	0.0261 (16)	0.0323 (18)	0.0249 (16)	−0.0131 (14)	0.0046 (13)	−0.0084 (14)
C20	0.0311 (18)	0.0330 (18)	0.0336 (18)	−0.0093 (15)	0.0087 (14)	−0.0164 (15)
C21	0.0340 (18)	0.0215 (16)	0.0354 (18)	−0.0024 (14)	0.0039 (14)	−0.0078 (14)
C22	0.0330 (18)	0.0260 (17)	0.0254 (16)	−0.0060 (14)	0.0028 (13)	−0.0043 (13)
C23	0.0234 (16)	0.0296 (18)	0.0252 (16)	−0.0051 (13)	0.0017 (12)	−0.0105 (14)
C24	0.0253 (16)	0.0261 (17)	0.0236 (16)	−0.0186 (14)	−0.0118 (13)	0.0098 (13)

### Geometric parameters (Å, °)

**Table:**

Cu1—O5	1.918 (2)	C5—H5	0.9500
Cu1—O1	1.928 (2)	C6—H6A	0.9900
Cu1—O9	1.949 (2)	C6—H6B	0.9900
Cu1—N1	2.002 (3)	C7—C8^i^	1.506 (5)
Br1—C11	1.887 (3)	C7—H7A	0.9900
Br2—C21	1.894 (3)	C7—H7B	0.9900
O1—C15	1.285 (4)	C8—C7^i^	1.506 (5)
O2—C15	1.230 (4)	C8—H8B	0.9900
O3—C16	1.284 (4)	C8—H8A	0.9900
O3—H3A	0.840 (19)	C9—C10	1.386 (5)
O4—C16	1.202 (4)	C9—C14	1.391 (5)
O5—C23	1.283 (4)	C9—C15	1.509 (4)
O6—C23	1.237 (4)	C10—C11	1.390 (5)
O7—C24	1.173 (4)	C10—H10	0.9500
O8—C24	1.265 (4)	C11—C12	1.385 (5)
O8—H8C	0.836 (19)	C12—C13	1.386 (5)
O9—H9A	0.827 (18)	C12—H12	0.9500
O9—H9B	0.824 (18)	C13—C14	1.388 (5)
N1—C5	1.334 (4)	C13—C16	1.517 (4)
N1—C1	1.351 (4)	C14—H14	0.9500
N2—C6	1.445 (5)	C17—C22	1.383 (4)
N2—C8	1.458 (4)	C17—C18	1.392 (4)
N2—C7	1.467 (4)	C17—C23	1.507 (4)
C1—C2	1.376 (5)	C18—C19	1.384 (4)
C1—H1	0.9500	C18—H18	0.9500
C2—C3	1.378 (5)	C19—C20	1.390 (5)
C2—H2	0.9500	C19—C24	1.554 (4)
C3—C4	1.377 (5)	C20—C21	1.380 (5)
C3—C6	1.507 (5)	C20—H20	0.9500
C4—C5	1.387 (5)	C21—C22	1.381 (5)
C4—H4	0.9500	C22—H22	0.9500
			
O5—Cu1—O1	176.53 (10)	C7^i^—C8—H8A	109.6
O5—Cu1—O9	89.58 (10)	H8B—C8—H8A	108.1
O1—Cu1—O9	90.21 (10)	C10—C9—C14	119.8 (3)
O5—Cu1—N1	90.77 (10)	C10—C9—C15	119.0 (3)
O1—Cu1—N1	90.64 (10)	C14—C9—C15	121.1 (3)
O9—Cu1—N1	159.57 (10)	C9—C10—C11	119.2 (3)
C15—O1—Cu1	120.4 (2)	C9—C10—H10	120.4
C16—O3—H3A	107 (3)	C11—C10—H10	120.4
C23—O5—Cu1	121.0 (2)	C12—C11—C10	121.3 (3)
C24—O8—H8C	115 (3)	C12—C11—Br1	119.8 (3)
Cu1—O9—H9A	122 (3)	C10—C11—Br1	118.9 (3)
Cu1—O9—H9B	117 (3)	C11—C12—C13	119.2 (3)
H9A—O9—H9B	112 (3)	C11—C12—H12	120.4
C5—N1—C1	117.1 (3)	C13—C12—H12	120.4
C5—N1—Cu1	121.3 (2)	C12—C13—C14	120.0 (3)
C1—N1—Cu1	121.5 (2)	C12—C13—C16	118.0 (3)
C6—N2—C8	111.4 (3)	C14—C13—C16	121.9 (3)
C6—N2—C7	111.6 (3)	C13—C14—C9	120.4 (3)
C8—N2—C7	109.6 (3)	C13—C14—H14	119.8
N1—C1—C2	122.6 (3)	C9—C14—H14	119.8
N1—C1—H1	118.7	O2—C15—O1	126.0 (3)
C2—C1—H1	118.7	O2—C15—C9	119.0 (3)
C1—C2—C3	120.1 (3)	O1—C15—C9	115.0 (3)
C1—C2—H2	119.9	O4—C16—O3	125.1 (3)
C3—C2—H2	119.9	O4—C16—C13	118.3 (3)
C4—C3—C2	117.4 (3)	O3—C16—C13	116.6 (3)
C4—C3—C6	121.3 (3)	C22—C17—C18	120.1 (3)
C2—C3—C6	121.4 (3)	C22—C17—C23	120.7 (3)
C3—C4—C5	119.8 (3)	C18—C17—C23	119.2 (3)
C3—C4—H4	120.1	C19—C18—C17	119.9 (3)
C5—C4—H4	120.1	C19—C18—H18	120.1
N1—C5—C4	122.9 (3)	C17—C18—H18	120.1
N1—C5—H5	118.6	C18—C19—C20	120.5 (3)
C4—C5—H5	118.6	C18—C19—C24	120.6 (3)
N2—C6—C3	111.0 (3)	C20—C19—C24	118.8 (3)
N2—C6—H6A	109.4	C21—C20—C19	118.5 (3)
C3—C6—H6A	109.4	C21—C20—H20	120.8
N2—C6—H6B	109.4	C19—C20—H20	120.8
C3—C6—H6B	109.4	C20—C21—C22	122.0 (3)
H6A—C6—H6B	108.0	C20—C21—Br2	119.7 (2)
N2—C7—C8^i^	109.4 (3)	C22—C21—Br2	118.3 (3)
N2—C7—H7A	109.8	C21—C22—C17	119.1 (3)
C8^i^—C7—H7A	109.8	C21—C22—H22	120.5
N2—C7—H7B	109.8	C17—C22—H22	120.5
C8^i^—C7—H7B	109.8	O6—C23—O5	125.5 (3)
H7A—C7—H7B	108.2	O6—C23—C17	119.3 (3)
N2—C8—C7^i^	110.3 (3)	O5—C23—C17	115.2 (3)
N2—C8—H8B	109.6	O7—C24—O8	128.0 (3)
C7^i^—C8—H8B	109.6	O7—C24—C19	118.5 (3)
N2—C8—H8A	109.6	O8—C24—C19	113.5 (3)
			
O9—Cu1—O1—C15	−79.5 (2)	C12—C13—C14—C9	−0.3 (5)
N1—Cu1—O1—C15	120.9 (2)	C16—C13—C14—C9	178.6 (3)
O9—Cu1—O5—C23	77.0 (2)	C10—C9—C14—C13	0.6 (5)
N1—Cu1—O5—C23	−123.5 (2)	C15—C9—C14—C13	179.8 (3)
O5—Cu1—N1—C5	19.0 (3)	Cu1—O1—C15—O2	3.3 (5)
O1—Cu1—N1—C5	−157.8 (3)	Cu1—O1—C15—C9	−176.2 (2)
O9—Cu1—N1—C5	109.9 (3)	C10—C9—C15—O2	−1.7 (5)
O5—Cu1—N1—C1	−162.0 (3)	C14—C9—C15—O2	179.1 (3)
O1—Cu1—N1—C1	21.2 (3)	C10—C9—C15—O1	177.9 (3)
O9—Cu1—N1—C1	−71.1 (4)	C14—C9—C15—O1	−1.4 (4)
C5—N1—C1—C2	0.2 (5)	C12—C13—C16—O4	3.3 (5)
Cu1—N1—C1—C2	−178.8 (3)	C14—C13—C16—O4	−175.6 (3)
N1—C1—C2—C3	1.4 (6)	C12—C13—C16—O3	−178.3 (3)
C1—C2—C3—C4	−2.2 (6)	C14—C13—C16—O3	2.8 (5)
C1—C2—C3—C6	178.9 (4)	C22—C17—C18—C19	0.6 (5)
C2—C3—C4—C5	1.4 (5)	C23—C17—C18—C19	−178.4 (3)
C6—C3—C4—C5	−179.7 (3)	C17—C18—C19—C20	−0.7 (5)
C1—N1—C5—C4	−1.0 (5)	C17—C18—C19—C24	179.2 (3)
Cu1—N1—C5—C4	178.0 (3)	C18—C19—C20—C21	0.1 (5)
C3—C4—C5—N1	0.2 (5)	C24—C19—C20—C21	−179.7 (3)
C8—N2—C6—C3	68.9 (4)	C19—C20—C21—C22	0.5 (5)
C7—N2—C6—C3	−168.3 (3)	C19—C20—C21—Br2	179.0 (2)
C4—C3—C6—N2	−129.7 (4)	C20—C21—C22—C17	−0.5 (5)
C2—C3—C6—N2	49.1 (5)	Br2—C21—C22—C17	−179.0 (2)
C6—N2—C7—C8^i^	177.1 (3)	C18—C17—C22—C21	0.0 (5)
C8—N2—C7—C8^i^	−59.0 (4)	C23—C17—C22—C21	179.0 (3)
C6—N2—C8—C7^i^	−176.4 (3)	Cu1—O5—C23—O6	0.1 (5)
C7—N2—C8—C7^i^	59.5 (5)	Cu1—O5—C23—C17	180.0 (2)
C14—C9—C10—C11	−0.3 (5)	C22—C17—C23—O6	179.4 (3)
C15—C9—C10—C11	−179.5 (3)	C18—C17—C23—O6	−1.6 (5)
C9—C10—C11—C12	−0.3 (5)	C22—C17—C23—O5	−0.4 (4)
C9—C10—C11—Br1	179.9 (2)	C18—C17—C23—O5	178.6 (3)
C10—C11—C12—C13	0.6 (5)	C18—C19—C24—O7	−176.8 (3)
Br1—C11—C12—C13	−179.6 (2)	C20—C19—C24—O7	3.1 (4)
C11—C12—C13—C14	−0.3 (5)	C18—C19—C24—O8	2.5 (4)
C11—C12—C13—C16	−179.2 (3)	C20—C19—C24—O8	−177.6 (3)

Symmetry codes: (i) −*x*+2, −*y*+1, −*z*+1.

### Hydrogen-bond geometry (Å, °)

**Table:**

*D*—H···*A*	*D*—H	H···*A*	*D*···*A*	*D*—H···*A*
O3—H3A···O7^ii^	0.84 (2)	1.98 (3)	2.763 (4)	155 (4)
O8—H8C···O4^iii^	0.84 (2)	1.81 (2)	2.629 (4)	166 (4)
O9—H9A···O2^iv^	0.83 (2)	1.82 (2)	2.635 (3)	169 (4)
O9—H9B···O6^iv^	0.82 (2)	1.83 (2)	2.647 (3)	171 (4)

Symmetry codes: (ii) *x*−1, *y*, *z*+1; (iii) *x*+1, *y*, *z*−1; (iv) −*x*, −*y*+2, −*z*.
